# Selectable encapsulated cell quantity in droplets via label-free electrical screening and impedance-activated sorting

**DOI:** 10.1016/j.mtbio.2023.100594

**Published:** 2023-02-28

**Authors:** Jianwei Zhong, Minhui Liang, Qiang Tang, Ye Ai

**Affiliations:** a Pillar of Engineering Product Development, Singapore University of Technology and Design, 8 Somapah Road, Singapore, 487372, Singapore; b Jiangsu Provincial Engineering Research Center for Biomedical Materials and Advanced Medical Devices, Faculty of Mechanical and Material Engineering, Huaiyin Institute of Technology, Huaian, 223003, China

**Keywords:** Single-cell encapsulation, Impedance-activated droplet sorting, Droplet microfluidics, Label-free analysis, Acoustofluidics

## Abstract

Single-cell encapsulation in droplets has become a powerful tool in immunotherapy, medicine discovery, and single-cell analysis, thanks to its capability for cell confinement in picoliter volumes. However, the purity and throughput of single-cell droplets are limited by random encapsulation process, which resuts in a majority of empty and multi-cells droplets. Herein we introduce the first label-free selectable cell quantity encapsulation in droplets sorting system to overcome this problem. The system utilizes a simple and reliable electrical impedance based screening (98.9% of accuracy) integrated with biocompatible acoustic sorting to select single-cell droplets, achieving 90.3% of efficiency and up to 200 ​Hz of throughput, by removing multi-cells (∼60% of rejection) and empty droplets (∼90% of rejection). We demonstrate the use of the droplet sorting to improve the throughput of single-cell encapsulation by ∼9-fold compared to the conventional random encapsulation process.

## Graphical abstract

**Figure:**
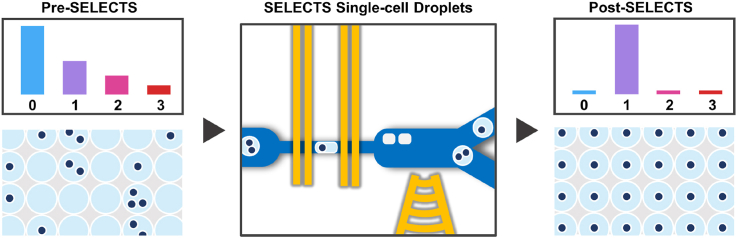

## 1 Introduction

Droplet microfluidics has become a promising technique in many biological and clinical applications, such as cell biology [1,2], disease diagnosis [3,4], and drug screening [5,6]. Single-cell encapsulation in droplets is particularly attractive as it enables profound studies for the behaviours of individual cells by reducing cross-contamination and absorption of chemicals, while maintaining cell activity and functionality at the single-cell level [7]. However, cell quantity encapsulated in droplets is a random process dominated by a Poisson distribution [8], resulting in a low encapsulation rate of single cells. Increasing droplet size and cell concentration in suspension can improve the encapsulation rate but yield more droplets encapsulating multi-cells. Inertial-based single-cell encapsulation methods are reported to overcome the limitation of a Poisson distribution by improving the individual cell encapsulation efficiency to about 80% [9,10]. Active single-cell encapsulation further enhances the single-cell encapsulation rate by over 90% [11,12] and demonstrates the high flexibility of integration to other downstream applications, including single-cell RNA sequencing [13], digital polymerase chain reaction [14] and biological assays [15].

Despite the superiorities of the current single-cell encapsulation approaches, there are still problems to be solved. Firstly, the efficiency of single-cell encapsulation dominates the throughput and purity of single-cell droplets. Elevating single-cell encapsulation efficiency increases the production throughput of single-cell droplets but could also introduce more multi-cells encapsulation in droplets due to the Poisson distribution. Current active sorting approaches constrain the probability of generating multi-cells droplets by manipulating the Poisson distribution that reduces cell concentration in suspension and droplet size [[16], [17], [18]]. However, they suffer from a rising number of empty droplets and diminishment of the probability of individual cell encapsulation, showing a trade-off relationship between the purity and throughput in single-cell encapsulation. Although some recent studies have demonstrated the differentiation between empty droplets and cell-encapsulated droplets [[19], [20], [21], [22]], none report the capability of multi-cells droplets removal to our best knowledge. Thus, the inherent limitation of the Poisson distribution becomes the key challenge that prohibits the further improvement of single-cell encapsulation efficiency in current active sorting techniques. Secondly, fluorescence labelling is widely employed in active droplet sorting [3,16,17,[23], [24], [25]], for accomplishing high accuracy on identifying cell-in-droplets. Label-free approaches that avoid cytotoxicity, bio-incompatibility, and non-specific binding from fluorophores in viable cell analysis [26,27], have been reported recently based on image-activated sorting [[28], [29], [30]]. Nevertheless, most depend on either complicated optical systems (reduction in system robustness and cost raises) or high computational power, such as machine learning (limitation on throughput, i.e., less than 4 ​Hz).

This work presents a SELECTS (SElectable Label-free Encapsulated Cell-in-dropleT Sorting) system, an electrical impedance-based microfluidic droplet sorter, to overcome the aforementioned trade-off by enabling not only empty droplet removal, but also the rejection of multi-cells droplets. It selects single-cell droplets regardless of the limitation of a Poisson distribution to achieve high purity and throughput. The SELECTS system employs two pairs of microelectrodes for sensing and determining the number of cells that are encapsulated in a droplet in a label-free manner and selectively activates a piezoelectric actuator to manipulate target droplets. With the integration of the flow-focused droplet generation structure, the SELECTS system delivers a direct approach that obtains high efficiency of single-cell encapsulated droplets by simply loading on the cell suspension. Here, the SELECTS system demonstrates high accuracy in differentiating empty and cell-encapsulated droplets (98.9%) via electrical impedance screening validated by high-speed imaging. With the high accuracy of impedance screening, the SELECTS system enriches single-cell encapsulated droplets to over 90%, while rejecting about 60% of multi-cells droplets. In addition to selecting single-cell droplets, the SELECTS system also demonstrates an opposite enrichment of multi-cells encapsulated droplets to about 16-fold, enabling the novel feature of selectable cell quantity encapsulated in droplets according to applications. Furthermore, we compare the throughput of single-cell droplets between low cell concentration ($10^{5}$ cells/ml), removing multi-cells droplets by manipulating the Poisson distribution, and high cell concentration ($10^{7}$ cells/ml) regardless of the probability of multi-cells encapsulation, and demonstrate the above 9-fold enhancement of the throughput for high cell concentration of suspension without degeneration of single-cell encapsulation efficiency. By voiding the limitation of Poisson distribution that freely selects droplets encapsulating specific cell quantity, the SELECTS system offers a novel solution with high purity and high throughput of customized cell number encapsulation for wide downstream applications in droplet microfluidics.

## 2 Materials and methods

### 2.1 Measurement on dielectric properties of cell-encapsulation droplet

**Fig. 1:**
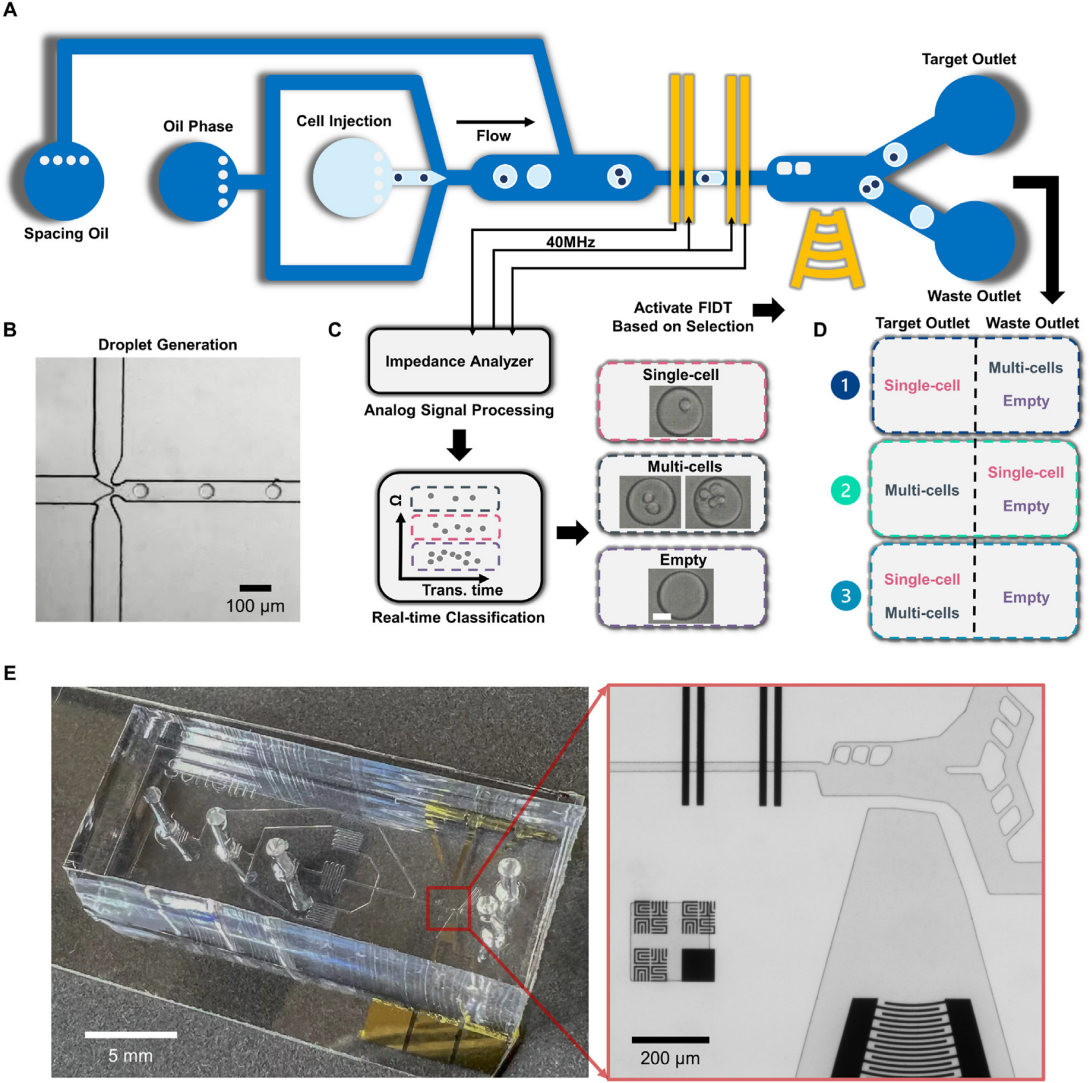
Overview of the SELECTS system. (**A**) Schematic of the SELECTS with the aqueous phase (light blue), oil phase (dark blue), electrode and FIDT (yellow). (**B**) Droplet generation in the SELECTS. (**C**) Principle of electrical impedance-based droplet screening. (**D**) Selectable combinations of droplets with various cell numbers in droplets. (**E**) Pictures of the constructed SELECTS.

The impedance analyser is an integration of a lock-in amplifier integrated with a microprocessor (HF2LI, Zurich instrument) and differential current amplifier (HF2TA, 1 ​kΩ gain). The lock-in amplifier generates 5 ​V at the frequency of 40 ​MHz to the central electrodes of the impedance sensing region (Fig. 1A). Choosing the high probe frequency is to effectively short the electric double layer and thin oil layer of droplets. 40 ​MHz probe frequency can also short the cell membrane in droplets so that the cytoplasm conductivity can be measured accurately, thus differentiating cell-encapsulated droplets. The microprocessor digitizes the analog impedance signals and finds the peak of the signal. Once target droplets are captured, an electrical pulse signal sends to activate a radio frequency (RF) generator for droplet sorting.

### 2.2 Acoustic radiation force acting on cell-encapsulation droplet and setting

A traveling acoustic wave scatters at the solid-liquid interface, producing a forward time-averaged acoustic radiation force (ARF, $F_{ARF}$) following the direction of wave propagation. The acoustic pressure can be expressed as:

Where $Y_{T}$ is the ARF factor dominated by density, *r* is the radius of the manipulated particle, and *E* is the average energy density of propagating wave. The calculation of $Y_{T}$ has been illustrated in our previous works [31]. The results in Fig. 4A indicate that $Y_{T}$ of droplets is basically unity after 10 ​μm in dimeters, and about 3 orders of magnitude larger than MCF-7. Thus, the ARF acting on droplets is independent of droplet content.

In experiments, after receiving the activation signal from the impedance analyser, the RF generator gives an RF signal at the resonance frequency of 132 ​MHz (acoustic wavelength of 30 ​μm) with acoustic power about 50 ​mW after RF amplification.

### 2.3 Fabrication of the SELECTS biochip

The micropattern for the poly(dimethylsiloxane) (PDMS) microfluidic channels was lithographed in SU-8 negative photoresist with 20 ​μm thickness. The impedance electrodes and FIDT were printed by electric-beam deposition on a 128 Y-cut X-propagation ${LiNbO}_{3}$ substrates with 100 ​nm thickness. The PDMS microfluidic channel with a 10:1 ​wt ratio of the prepolymer and the curing agent was bonded to a well-patterned ${LiNbO}_{3}$ substrate after plasma treatment. The chip is then baked in a 60 ​C oven overnight for further experiments.

### 2.4 Cell sample culture and preparation

MCF-7 ​cells (ATCC cat. no. HB-7) were cultured under the standard protocol reported in our previous studies [32]. The trypsinized MCF-7 ​cells were resuspended to a low conductive buffer (BTXPRESS Low Conductivity Medium T, BTX, USA). The low conductive buffer is biocompatible as it maintains the osmotic pressure at ∼270 mOsm/L, and is nontoxic according to the manufacturer's datasheet. The permeabilized MCF-7 ​cells were heat-treated at 60 ​C for 10 ​min, and then fixed with 4% formaldehyde. After permeabilization, MCF-7 ​cells were stained with trypan blue for 5 ​min and washed with the low conductive buffer three times.

### 2.5 Evaluation of the viability of MCF-7 ​cells encapsulated in droplets

The fluorinated oil was stored in a cell incubation at 37 ​°C and 5% ${CO}_{2}$ for overnight. Then the sorted and unsorted MCF-7 ​cells droplets were collected and sealed in a 1 ​ml tubes to prevent evaporation. Every 3 ​h, we transferred 10 ​μl of droplets to 200 ​μl tubes and recover cells in droplets by adding an emulsion breaker (Fluoro-Stop, Dolomite Microfluidics) in 1:1 ​vol ratio. The cell viability is determined by using trypan blue staining.

### 2.6 Operation of the SELECTS system

Before starting sorting experiments, the oil phase and spacing phase (fluorinated oil, Novec 7500, 3 ​M) with 2% (w/w) FluoSurf (Dolomite, UK) and aqueous phase of cell suspension in a low conductive buffer are loaded to the SELECTS system. With a stable generation of droplets (40 ​μm in diameters), sampling the impedance signals of droplet mixtures for 1 ​min is performed for setting an appropriate impedance gating interval to select single-cell droplets (Fig. S1). After loading the impedance gate in the controlled program in the microprocessor of the impedance analyser, the sorting process, a cooperation of an embedded program and hardware, starts as illustrated in Fig. S2. The impedance demodulator in the impedance analyser and the embedded program continuously communicates the real-time impedance signals to find peaks. The embedded program determines the types of droplets and activates digital-input-output in the impedance analyser by setting to high voltage. Once activating the RF signal generator, a pre-set RF signal is generated and amplified for droplet manipulation through FIDT.

### 2.7 Numerical simulation for electrical properties of cell-encapsulation droplet

The simulation was setup in the Electric Currents (ec) module in COMSOL 5.0 (USA) with 2D finite element modelling. The dimensional and electrical parameters [33] were summarized in Table S1 and shown in Fig. S3. The fluidic model was confined in a rectangular region with a length of 180 ​μm and a width of 20 ​μm. Two rectangular electrodes with a thickness of 100 ​nm were centered symmetrically with 20 ​μm in the bottom of the simulation region. The dielectric constant of carrier oil (HFE-7500, 3 ​M) is set to 5.8. In the simulation, the potential of the left electrode was 5 ​V, and the right one was grounded. The remaining boundaries of the fluidic region were set to electric isolation.

### 2.8 Statistical analysis

Data presented as mean with error bars of standard deviations is summarized from three experimental results. The statistical significance is evaluated by the two-samples *t*-test in MATLAB, where N.S. indicates the statistic not significant, ∗p ​< ​0.05, ∗∗∗p ​< ​0.001. At least over 700 droplets were counted for each analysis to provide statistical significance, otherwise specified.

## 3 Results and discussion

### 3.1 Working principle of the SELECTS system

As shown in Fig. 1A, the SELECTS system integrates three main parts, including droplet generation for cell encapsulation, impedance sensing for cell quantity in droplet determination, and focus integrated transducer (FIDT) for droplet manipulation. Droplets generated from a flow-focused structure (Fig. 1B) encapsulate a random quantity of cells based on cell concentration and droplet size and are screened by two pairs of electrodes to characterize their dielectric properties. The electrodes and microfluidic channel are designed and arranged specifically for screening droplets in diameters from 30 to 80 ​μm, defined as the impedance sensing region. The electrodes also cooperate as a differential configuration in that the measured impedance equals the subtraction of both pairs of electrodes to improve the sensitivity of droplet impedance measurement. When a droplet passes through the first pair of electrodes, the aqueous medium in droplets replaces the original non-conductive oil, causing changes of impedance that are characterized by the probe frequency (40 ​MHz). The differential impedance signals (Fig. S4) are then digitized and processed by an impedance analyser (Fig. 1C). Thanks to the high probe frequency, the AC current is able to permeabilize into the interior of droplets and characterize the encapsulated content of droplets. Because the optimized electrical conductivity of the aqueous medium is significantly inferior to that of cellular cytoplasm (∼5 orders of magnitude), the quantity of cell encapsulation can be concluded from the magnitude of the measured impedance for target droplets, including empty, single-cells, and multi-cells. A trigger signal is sent to activate the FIDT controlled by a customized embedded program. Therefore, based on the impedance classification of three categories of cell-encapsulation droplets, there are three options for customized selections that can be collected from the target outlet, including single-cell droplets, multi-cells droplets, and both (Fig. 1D). The SELECTS system is constructed to exhibit the ability to select droplets of various cell quantity encapsulation (Fig. 1E). The microchannel of impedance sensing region is designed to have a width of 30 ​μm and a height of 20 ​μm, to optimize the detection sensitivity but not squeeze encapsulated cells. A demonstration of integrating real-time impedance signals and sorting process (Movie S1) illustrates that the customized algorithm filters out the impedance signals of empty and multi-cells droplets by selecting only single-cell encapsulation droplets for the target outlet (up).

Supplementary data related to this article can be found at https://doi.org/10.1016/j.mtbio.2023.100594.

The following is the supplementary data related to this article:

Movie S1 Movie S1

### 3.2 Optimization and determination of single-cell encapsulation by dielectric properties

Electrical-based single-cell encapsulation droplet screening has demonstrated inherent expediencies of label-free and high-throughput in differentiating empty droplet and cell-encapsulation droplets [34]. However, few works deliver a comprehensive study of improving the sensitivity of electrical-based cell-in-droplet detection. To address this, we perform detailed simulations of cell-encapsulation droplets and construct an electrical-activated imaging system to optimize the detection of cell-encapsulation droplets (Fig. S5). When a droplet passes through the electrodes in the detection area, the impedance signals with a symmetric peaks feature is generated and trigger a high-speed camera as it reaches the pre-set threshold. The system can image 100% droplet events to verify individual impedance signals induced by corresponding droplets.

**Fig. 2:**
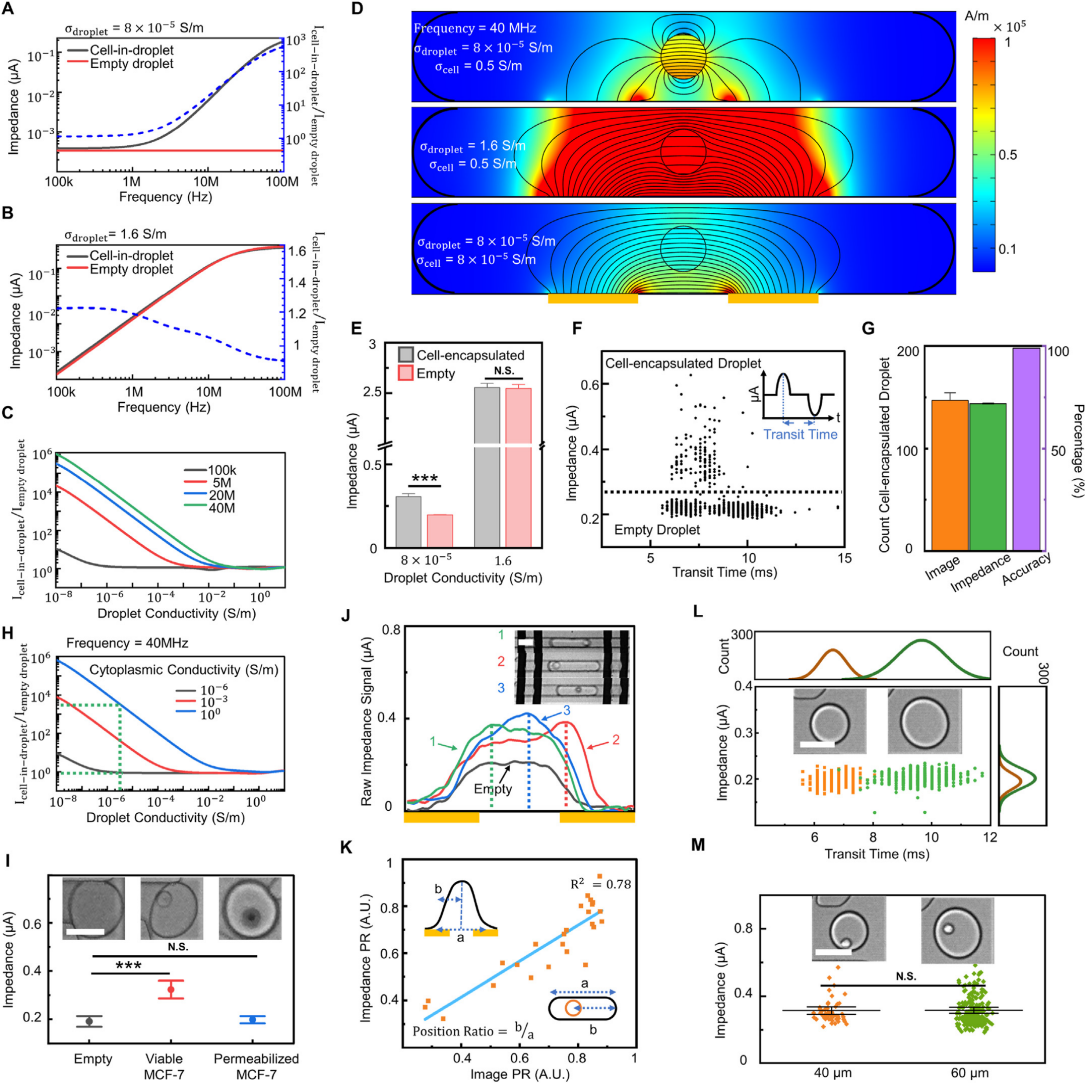
Single-cell encapsulation in droplets screening via electrical impedance approach and its optimization. (**A**–**B**) Simulation of probe frequency spectrum for low and high conductivity of aqueous medium in droplets. (**C**) Simulated conductivity spectrum of aqueous medium in droplet with probe frequencies of 100 ​kHz, 5 ​MHz, 20 ​MHz, and 40 ​MHz. (**D**) Simulation of electric field distribution and current density with different droplet and cell conductivity at 40 ​MHz. Note that the droplet boundaries have been blacked and bulked. (**E**) The impedance of empty and cell-encapsulated droplets verified by a high-speed camara. (**F**) The impedance of mixture droplets with a gate to differentiate empty and cell-encapsulated droplets. (**G**) Validation of imaging and impedance-based approaches with a high accuracy of 98.9%. (**H**) Simulation of droplet conductivity spectrum with different cytoplasm conductivity of encapsulated cells. (**I**) Impedance of viable cells encapsulated, permeabilized cells encapsulated and empty droplets with respective microscopic images. (**J**–**K**) The position of cell-in-droplets characterized by both impedance and imaging-based approaches and correlation. (**L**–**M**) Demonstrations of impedance invariance for both empty and cell-encapsulated droplets. Scale bar indicates 50 ​μm. Data are presented as mean ± s.d. (standard deviation). N.S. indicates statistic not significant, ∗∗∗p ​< ​0.001.

The simulation illustrates that differentiation between empty and cell-encapsulated droplets rises beyond 10-times with the increasing probe frequency (>10 ​MHz) at low conductive (${8\times 10}^{-5}$ S/m) of aqueous medium (Fig. 2A). In contrast, the sensitivity of discriminating cell-encapsulation droplets diminishes after 1 ​MHz with high conductive medium (i.e., phosphate-buffered saline buffers (PBS), conductivity ​= ​1.6 ​S/m) (Fig. 2B). Experimental results agree that increasing probe frequency in the low conductivity medium enhances the sensitivity of differentiating empty and cell-encapsulated droplets (Fig. S6). The simulation further suggests that lowering the electrical conductivity of the aqueous medium in droplets results in higher discrimination of empty and cell-encapsulation droplets (Fig. 2C). The presence of a highly conductive object (suspended single-cells with cytoplasm conductivity of 0.5 ​S/m) in the low conductive droplets vastly reduces the average conductivity of droplets compared to empty droplets (Fig. 2D top). However, due to the conductivity of PBS being higher than cell cytoplasm, the encapsulated cells are shorted by the surrounding PBS and become undetectable via the impedance-based approach (Fig. 2D mid). Experimental results at 40 ​MHz probe frequency (Fig. 2E) approve the conclusion drawn by simulations and demonstrate that the modified conductivity of an aqueous medium optimizes the sensitivity of impedance measurement of cell-encapsulated droplets, and enables direct gating from the mixtures of random encapsulation droplets with the accuracy of 98.9% (Fig. 2F and G). Furthermore, by varying cell diameters in the simulation, a high probe frequency (>10 ​MHz) could maintain a large impedance ratio between single-cell droplets and empty droplets regardless of cell diameters (Fig. S7). This indicates the potential of using SELECTS to distinguish droplets that encapsulate cells from not only different cell lines having similar diameter distribution (Fig. S8), but also different cell types in various cell diameters.

To further verify the conclusion, we study the cases of various cell cytoplasm conductivity and report that with more significant differences between cytoplasm and droplet conductivity, a higher detection sensitivity can be achieved (Fig. 2H). Experimental results illustrate a similar impedance magnitude for empty droplets and permeabilized MCF-7 ​cells (stained in trypan blue) encapsulated droplets, whereas viable MCF-7 ​cells encapsulated droplets remain significant difference from empty droplets (Fig. 2I). This is because permeabilized cell membrane allows exchange between high conductive cytoplasm and low conductive aqueous buffer resulting in encapsulated cells become undetectable by impedance analyser (Fig. 2D bottom). An interesting observation is that after the optimization of sensitivity in cell-encapsulation detection, the position of cell-in-droplets is strongly related to that of peaks in impedance signals ($R^{2}=0.78$) verified by the imaging system (Fig. 2J and K). In addition, experiments further reveal that the impedance magnitude is not affected by the size of droplets regardless of cell encapsulation (Fig. 2L and M). The droplet size can be determined by the transit time in the impedance screening region and the imaging system. The droplet-size insensitivity could potentially become an advantage for applications that could break the uniformity of droplet sizes, such as PCR during thermo-cycling.

### 3.3 Electrical detection of multi-cells encapsulated droplets

**Fig. 3:**
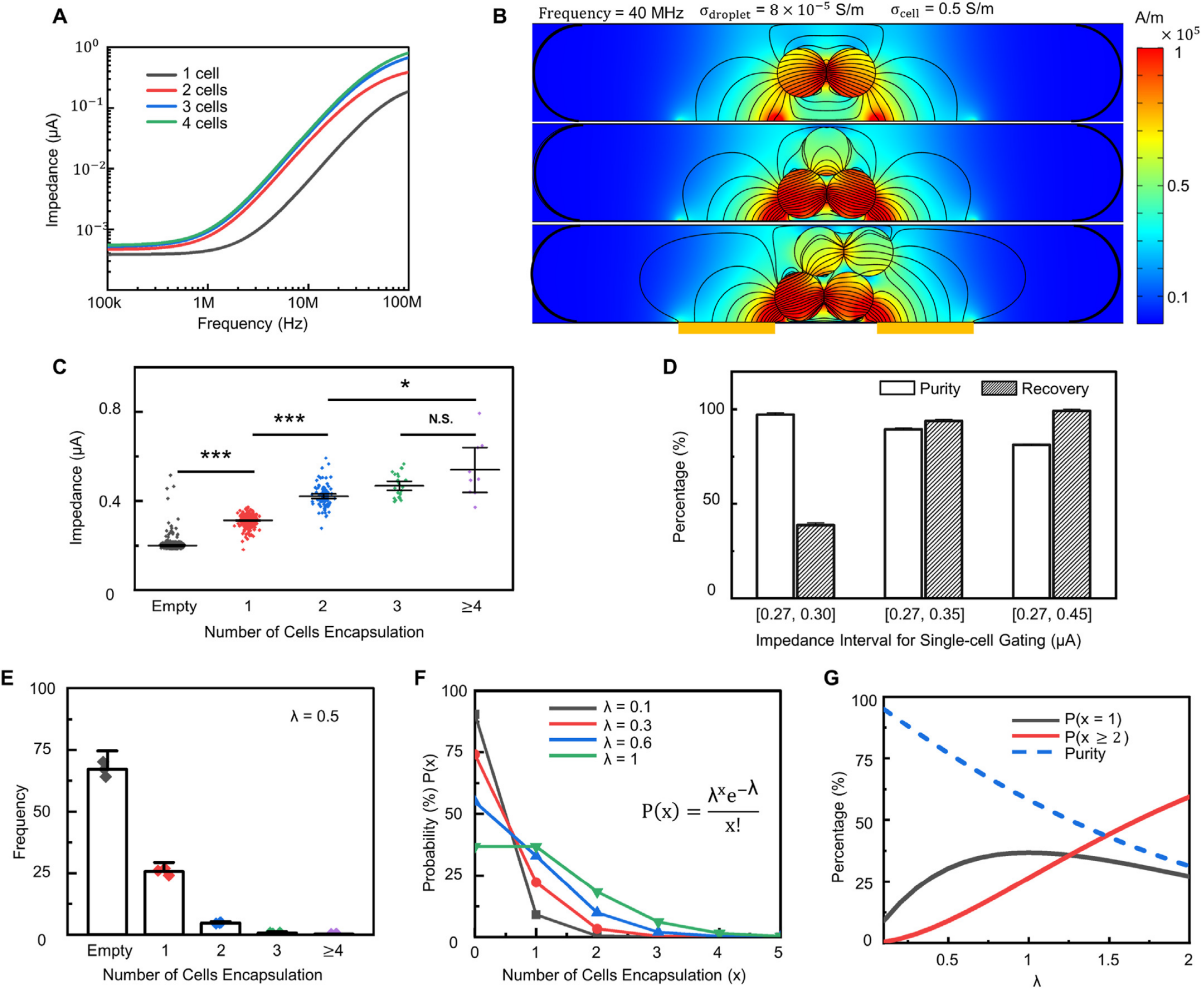
Differentiation between single-cell and multi-cells encapsulated droplets by electrical impedance approach. (**A**) Simulation of probe frequency spectrum for various cell numbers in droplets. (**B**) Simulation of electric field distribution and current density with different droplet and cell conductivity at 40 ​MHz. Note that the droplet boundaries have been blacked and bulked. (**C**) Impedance of different cell numbers in droplets validated by a high-speed camara. (**D**) Purity and recovery of various impedance gating intervals. (**E**) Experimental probability of cell-encapsulation efficiency of different cell numbers in droplets when **λ** ​= ​0.5. (**F**) Theoretical calculations of the probability for various cell numbers in droplets with different λ (≥ 0.1). (**G**) Illustrations of the corruption of purity for single-cell encapsulated droplets when increasing λ from 0 to 2 without multi-cells droplets removal. Data are presented as mean ± s.d (n ​= ​3). N.S. indicates statistic not significant, ∗p ​< ​0.05, ∗∗∗p ​< ​0.001.

The aforementioned trade-off indicates that the purity and throughput of single-cell encapsulation droplets could not improve simultaneously in random encapsulation, as reducing the probability of empty droplets leads to an increment of multi-cells encapsulation in droplets. To overcome the trade-off, we optimize the proposed device for discriminating multi-cells in droplets from single-cells. The simulated probe frequency spectrum of different cell numbers encapsulated in droplets illustrates that multi-cells droplets can be differentiated beyond 1 ​MHz (Fig. 3A) and demonstrates a higher magnitude of impedance to single-cell encapsulated droplets. The reason is that the average conductivity of droplets is further enhanced due to the encapsulation of multi-cell aggregates. In addition, multi-cell aggregates occupy larger electrical detection volume (the space above and between electrodes), resulting in a decrease in effective series resistance between cells and electrodes ($R_{Droplet\left(side\right)}$) (Fig. 3B and Fig. S9). However, it is challenging to identify droplets encapsulating more than one cell in the frequency range of 1–40 ​MHz, as the effective series resistance and the average conductivity in the electrical detection volume remain similar with more cells encapsulated in droplets. The experiment results from the electrical-activated imaging system further emphasize that the measured impedance of single-cell droplets is significantly smaller than the multi-cells encapsulation droplets, whereas it is a minor difference between two and more cells encapsulation (Fig. 3C). Therefore, by optimizing the gate of impedance magnitude, the purity, and recovery of single-cell encapsulation efficiency can be improved to both about 90% (Fig. 3D).

**Fig. 4:**
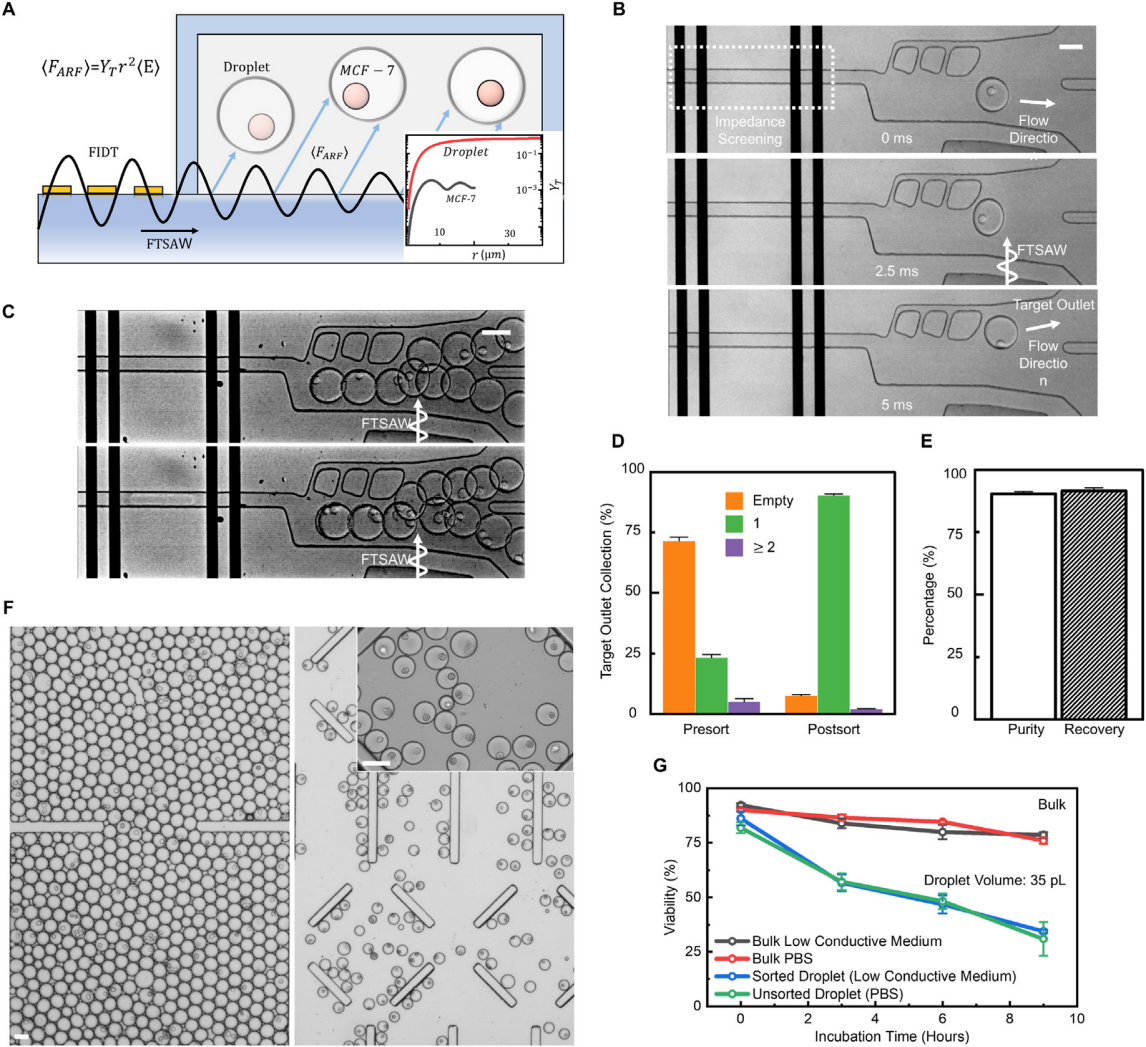
Demonstration of selecting single-cell encapsulated droplets by rejecting empty and multi-cells encapsulated droplets. (**A**) Illustration of acoustic radiation force generation and actuation on droplets for manipulation. The sub-plot on the right-bottom corner explains the acoustic radiation factor for droplet and cells (MCF-7). (**B**) The sorting processes for manipulating single droplets to the target outlet in 5 ​ms. (**C**) Demonstration of the SELECTS system selects single-cell droplets while removing empty (top) and multi-cells encapsulated droplets (bottom) by acoustic wave (FTSAW). (**D**–**E**) Illustration of the performance of SELECTS for selecting single-cells droplets with 90.3% purity by eliminating empty droplets (from 71.5% to 7.6%) and multi-cells encapsulated droplets (from 5.1% to 2.1%) with 91.5% recovery. (**F**) Bright-field images of unsorted and SELECTS sorted single-cell encapsulated droplets. (**G**) 9-h incubation of cells packed in sorted low conductive medium droplets, unsorted PBS droplets, suspension of low conductive medium and PBS. Note that the viability of MCF-7 ​cells incubated in bulk PBS and the low conductive medium is similar, indicating the low conductive medium is biocompatible. Data are presented as mean ± s.d. (n ​= ​3). Scale bar indicates 50 ​μm.

The λ in a Poisson distribution, defined as the fraction of the volume fraction of cells in the bulk suspension to that of a cell encapsulated in a droplet [8], controls the probability of single-cell droplets in random encapsulation. With the controlled cell concentration in suspension, the imaging system counts over 25% of single-cell encapsulation in droplets and about 6% of multi-cells droplets (Fig. 3E). The single-cell encapsulation probability can be improved with higher λ and reach the peak (36.7%) when λ ​= ​1. Thus, the throughput of single-cell droplets can be elevated at least 4-fold compared to low λ (≤ 0.1) adopted in conventional random single-cell encapsulation (Fig. 3F). However, the highest probability of single-cell encapsulation comes with about 26.4% of multi-cell encapsulation (λ ​= ​1) resulting in the theoretical purity (% ​= ​P(x ​= ​1)/P(x ​> ​0)) of single-cell encapsulation drops less than 60% without multi-cells droplet removal (Fig. 3G).

### 3.4 Selection of cell quantity in droplets via impedance-activated sorting

Selecting single-cell droplets becomes necessary to improve the purity at high λ (0.5, $10^{7}$ cells/ml). Unlike conventional dielectrophoretic force (DEP) sorting techniques due to its potential for biohazard from the high electric field, surface acoustic wave has an innate superiority of biocompatibility in droplet sorting due to the use of acoustic radiation force (ARF) that manipulates droplets gently (Fig. 4A). Notably, since the simulated ARF factor $Y_{t}$ of droplets shown in the subplot of Fig. 4A is 3 orders of magnitude larger than MCF-7 ​cells, the ARF acting on droplets predominates the manipulation process regardless of cell encapsulation (Movie S2). The SELECTS system is presented by integrating the acoustic sorting actuator (FIDT). When a droplet flows through the impedance screening region, it follows the flow direction to the waste outlet by default (Fig. 4B top) if the FIDT is not activated. On the other hand, single-cell encapsulated droplets can be detected by the impedance screening region and pushed by a focused traveling surface acoustic wave (FTSAW) produced by the activated FIDT resulting in a change of flow direction to the target outlet (Fig. 4B mid and bottom, see Movie S3).

Supplementary data related to this article can be found at https://doi.org/10.1016/j.mtbio.2023.100594.

The following are the supplementary data related to this article:

Movie S2 Movie S2

Movie S3 Movie S3

By employing the aforementioned impedance gating, the SELECT system demonstrates the capability of rejecting both empty droplets and multi-cells droplets (Fig. 4C and Movie S4). Eventually, the single-cell encapsulation efficiency can be improved to 90.3% while diminishing the ratio of multi-cells from 5.1% to 2.1% while maintaining over 90% of the recovery rate from the SELECT system (Fig. 4D and E). Fig. 4F further demonstrates the sorting results in microscopic images compared with the unsorted sample. Note that droplet manipulation via FTSAW shows biocompatible to cells encapsulated in droplets with similar cell viability to unsorted droplets, which is demonstrated in 9-h droplet-based cultivation (Fig. 4G). Another observation is that cell viability diminishes faster when encapsulating in droplets than in bulk cultivation, as agreed by a previous study [11,35]. This emphasizes the importance of minimizing the processing time of cell-in-droplets since dead cells could be destructive to downstream applications (i.e., single cell RNA sequencing).

Supplementary data related to this article can be found at https://doi.org/10.1016/j.mtbio.2023.100594.

The following is the supplementary data related to this article:

Movie S4 Movie S4

**Fig. 5:**
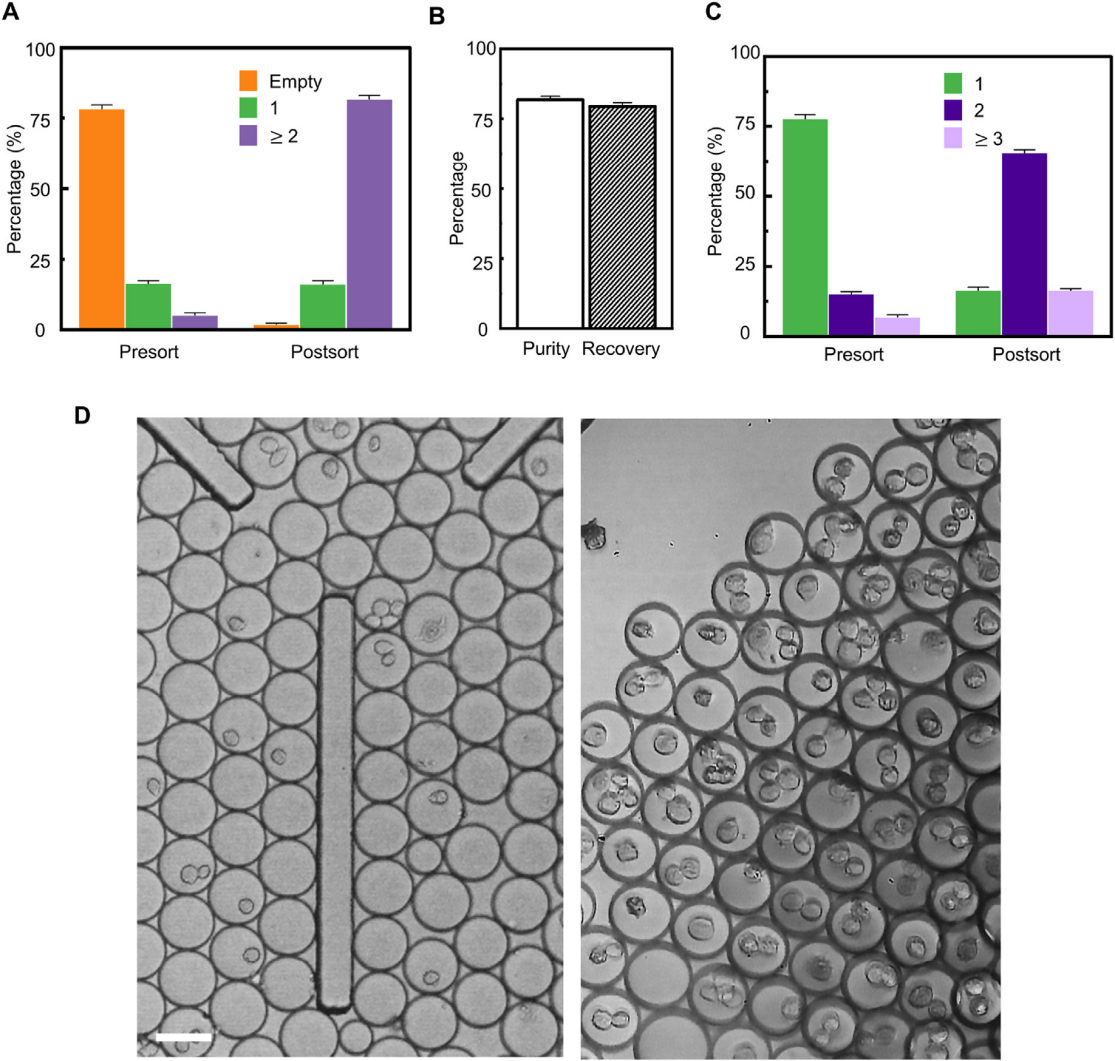
Demonstration of selecting multi-cells encapsulated droplets by rejecting empty and single-cell encapsulated droplets. (**A**–**B**) Illustration of the performance of SELECTS for selecting multi-cells droplets with enrichment from 5.2% to 81.7% with 79.3% recovery. (**C**) Performance of SELECTS that enriching co-encapsulated cells (purple) in droplets from 15.3% to 65.6% while rejecting single-cell droplets from 77.8% to 16.6% among all cell-encapsulated droplets. (**D**) Bright-field images of unsorted and SELECTS sorted multi-cells encapsulated droplets. Data are presented as mean ± s.d. (n ​= ​3). Scale bar indicates 50 ​μm.

In addition to single-cell droplet selection, the SELECT system is able to deliver multi-cells droplets by rejecting empty droplets and single-cell droplets as well. With the impedance gate is set to above 0.4 ​μA, the SELECT system reversibly enriches about 16-fold of the multi-cells encapsulation efficiency (81.7%) with the recovery of about 80% (Fig. 5A and B). The dual-cells droplets have been enriched from 15.3% to 65.6% while rejecting single-cell droplets from 77.8% to 16.6% (Fig. 5C). Fig. 5D further demonstrates the microscopic images of sorting results of multi-cells encapsulation in droplets. Since a Poisson distribution dominates the number of cell encapsulated in droplets, dual-cells encapsulation efficiency can be further improved by manipulating λ for applications such as cell fusion [36] and antigen-presenting cells activation [2,23,37].

### 3.5 Throughput comparison with conventional low λ in single-cell encapsulation

**Fig. 6:**
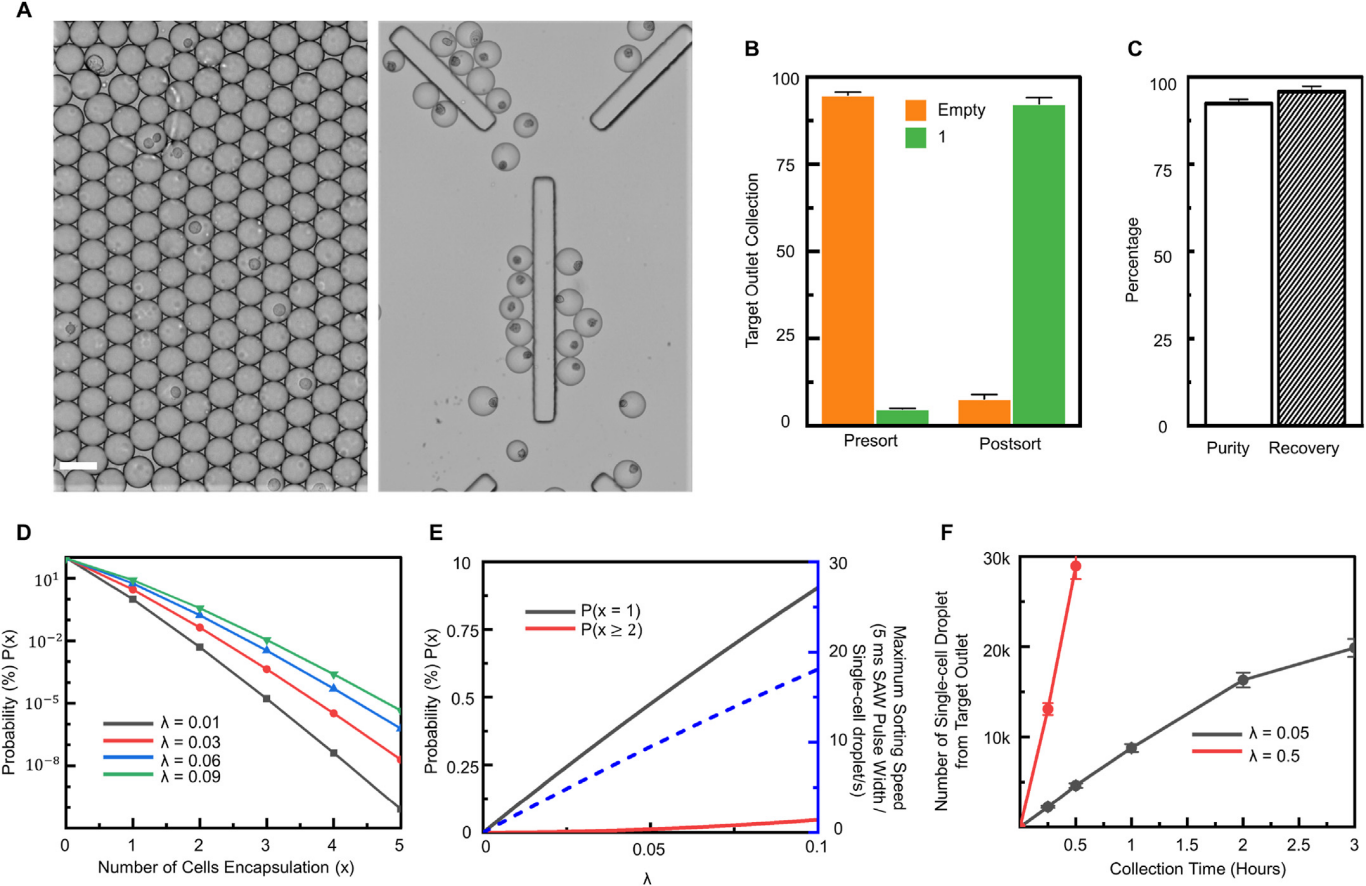
Throughput comparison between high λ (0.5) and low λ (0.05) in a random single-cell encapsulation. (**A**) Bright-field images of unsorted and SELECTS sorted single-cell encapsulated droplets when λ ​= ​0.05. (**B**–**C**) Illustration of the performance of SELECTS for selecting single-cells droplets with enrichment from 4.8% to 92.3% with 95.8% recovery. (**D**) Theoretical calculations of the probability for various cell numbers in droplets with different λ (≤ 0.1). (**E**) Illustration of theoretical throughput limitation for low λ with an assumption of maximum throughput is 200 ​Hz. (**F**) Experimental throughput comparison of a collection of 28,948 single-cell encapsulated droplets for high λ (0.5) and 19,875 single-cell encapsulated droplets for low λ (0.05). Data are presented as mean ± s.d. (n ​= ​3). Scale bar indicates 50 ​μm.

Conventional droplet sorting techniques adopt low λ ($10^{5}$ cells/ml) for limiting the probability of multi-cells encapsulation in order to achieve high purity of single-cell droplets. By reducing the cell concentration in suspension to $10^{5}$ cells/ml and setting λ ​= ​0.05, the multi-cells encapsulation rate drops to less than 0.5%, with the single-cell encapsulation efficiency of 4.8% (see Fig. 6A left). The SELECTS system maintains the capability of enriching single-cell droplets to 92.3% with 95.8% recovery in low cell concentration samples (Fig. 6A right, B, and C). Although it is less concerned about the destruction of single-cell encapsulation purity when reducing λ less than 0.05 (the theoretical probability of multi-cells encapsulation drops to less than 0.2%), the theoretical maximum throughput of single-cell droplets is inhibited to less than 10 droplets/second with current acoustic setting (Fig. 6D and E). Therefore, the throughput of single-cell droplets is critically limited by low λ due to the deficient probability of single-cell encapsulation. By comparing the actual throughput of when λ ​= ​0.5 and 0.05, the higher λ contributes about 30 ​k single-cell droplets in 30 ​min, whereas the lower λ can only provide less than 20 ​k single-cell droplets in 3 ​h (Fig. 6F). Notably, the speed of droplet generation declines after 2 ​h due to the droplet merge and size variation for keeping a long time in an on-chip queuing chamber of the SELECTS. Furthermore, a prolonged sorting time in hours decreases cell viability in water-oil droplets dramatically (∼25% in 3-h droplet-based cultivation discussed in Fig. 4G); therefore, a high λ could ultimately shorten the sorting time to about 30 ​min with the throughput of 30 ​k cells and only cost less than 5% cell viability loss. In addition to cell viability, a high probability (>90%) of generating empty droplets that encapsulate and waste reagents for cell analysis in low λ (<0.1), brings cost issues to expensive applications such as single-cell RNA sequencing, which can be solved by the SELECTS with high λ cell suspension.

## 4 Conclusion and outlooks

The proposed SELECTS system combines the ascendency of label-free high-throughput electrical screening with biocompatible acoustic sorting in a microfluidic chip, overcoming a decisive limitation of throughput and purity for single-cell encapsulated droplets in previous microfluidic active droplet sorting techniques. The proposed system achieves 90.3% of purity and up to 200 ​Hz of throughput for single-cell encapsulation in droplets by removing ∼90% of empty droplets and ∼60% of multi-cells droplets from high λ (0.5) random cell capsulation. Rejecting multi-cells encapsulated droplets is a novel breakthrough of Poisson distribution in random single-cell encapsulation that can ultimately elevate the throughput only dependent on the sorting speed of devices. The capability of co-encapsulation of cells in droplets (∼16-fold enrichment) can be further utilized in cell-to-cell interaction for next-generation cancer immunotherapy, such as biological assay for co-encapsulation of T cells and tumor cells [23,24]. Other downstream applications can be easily integrated into the SELECTS system, such as barcode-based single-cell PCR. Since high λ results in fewer empty droplets, barcode particles can be effectively co-encapsulated with cells in droplets, unlike conventional techniques, most of them are wasted in empty droplets. In addition, this work gives insight to future studies of cell-encapsulation in droplets that dielectric properties of cell-in-droplets could be a favorable solution due to the feature of label-free in the SELECTS system with similar accuracy to conventional staining approaches. It prevents interference of normal cell behaviours and functions from fluorescent labelling that offer a new revenue to droplet-based biological analysis such as autofluorescence, cell-cycling, and cell functionality research [[38], [39], [40]].

The capabilities of the SELECTS system can be further extended in several directions. Firstly, the SELECTS is potentially applicable to various cell types, in addition to MCF-7 ​cell lines, because distinguishing droplets encapsulating cells is based on the electrical conductivity difference between cellular cytoplasm and the in-droplet aqueous solution. Since most of the cellular cytoplasm [41] has the conductivity that is several orders of magnitude higher than the low conductive medium, the SELECTS has a great potential to be utilized on different cell types. Secondly, differentiating viable and permeabilized cells encapsulation in droplets has been demonstrated in Fig. 2, but viability enrichment of viable-cell encapsulation in droplets has yet to be shown. Thus, further work can be done on delivering viable single-cell droplets that could be particularly useful for single-cell RNA sequencing. Thirdly, impedance-based approaches that have been used for differentiating cell types based on cellular morphologies (i.e., cell size, intracellular complexity, and cell dielectric and mechanical properties) in previous studies [33,42,43], may integrate into droplet microfluidics, achieving cell subtypes discrimination in droplets for high sensitivity of sing-cell analysis. To implement these applications, the performance of the SELECTS system should be further improved, such as optimizations of impedance sensing electrodes for identifying precise cell numbers encapsulated in droplets, and the throughput by reducing acoustic pulse width. With these improvements, the SELECTS system has the potential for paving the revenue of droplet microfluidics in a broad range of biological and medical applications that have not been feasible due to the roadblock of Poisson distribution.

## Author contributions

Y.A. conceived the concept of the SELECTS system. J.Z. designed and constructed the SELECTS system. J.Z. and M.L. carried out the electrical-based imaging system. J.Z. analysed and visualized experimental data. J.Z. and Q.T. performed numerical simulations. J. Z wrote the first draft of manuscript. Y.A., J.Z. and M.L revised and edited the manuscript. All authors approved the final version of the article.

## Data and materials availability

All data to evaluate the conclusions in the paper are present in the paper and the Supplementary.

## Declaration of competing interest

The authors declare the following financial interests/personal relationships which may be considered as potential competing interests: Ye Ai has co-founded a start-up company, CellWave Technologies Pte. Ltd., to commercialize the acoustic sorting technology.

## Data Availability

Data will be made available on request.
