# Supplementary material for: Selectable encapsulated cell quantity in droplets via label-free electrical screening and impedance-activated sorting
Source: Mater Today Bio. 2023 Feb 28;19:100594. doi: 10.1016/j.mtbio.2023.100594 (PMC9999206; doi:10.1016/j.mtbio.2023.100594)
Supplement: Multimedia component 5 [file mmc5.pdf]

Supplementary Materials for

Selectable encapsulated cell quantity in droplets via label-free electrical screening and impedance-activated sorting

Jianwei Zhong[†,1], Minhui Liang[†,1], Qiang Tang[2], and Ye Ai[*,1]

[1] Pillar of Engineering Product Development, Singapore University of Technology and Design, 8 Somapah Road, Singapore 487372, Singapore

[2] Jiangsu Provincial Engineering Research Center for Biomedical Materials and Advanced Medical Devices, Faculty of Mechanical and Material Engineering, Huaiyin Institute of Technology, Huaian, 223003, China

† These authors contributed equally to this work.
*Corresponding author. Email: aiye@sutd.edu.sg; Tel: (+65) 6499 4553

This PDF file includes:

Fig. S1 to S9
Tables S1
Movie S1 to S4

Other Supplementary Materials for this manuscript include the following:
Movie S1 to S4

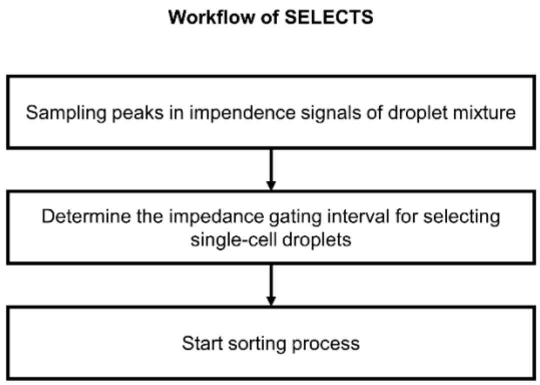

Fig. S1. General workflow of the SELECTS system. This workflow is standardized for repeatability and reliability of SELECTS in experiments.

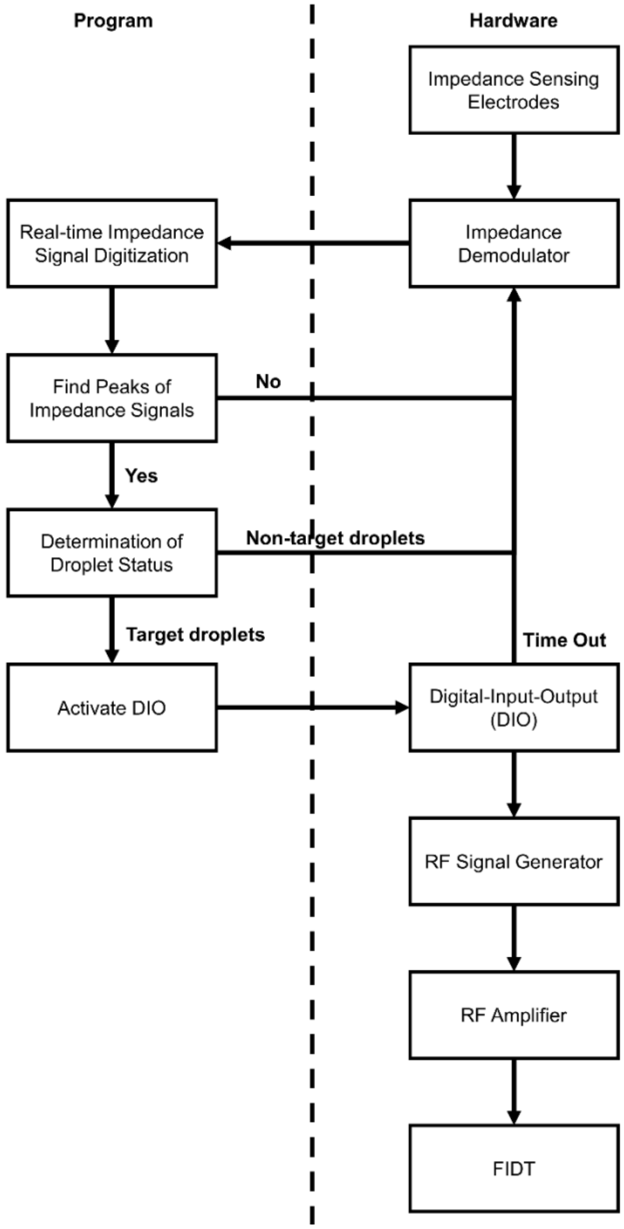

Fig. S2. Workflow of the electrical system of the SELECTS. This illustrates the cooperation (signal path) of software (control program) and hardware of the SELECTS system.

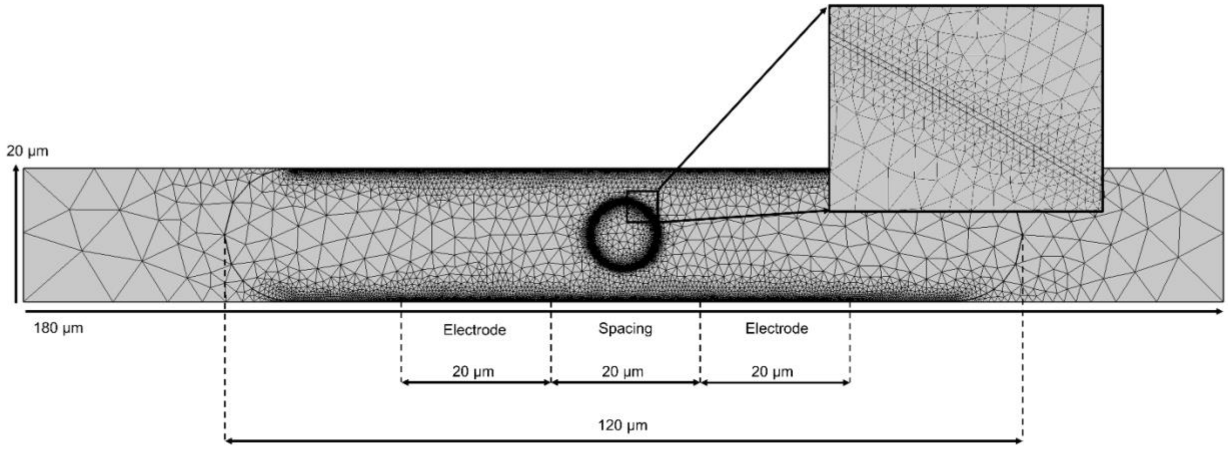

Fig. S3. Finite element modeling of single-cell encapsulation in droplets in COMSOL.

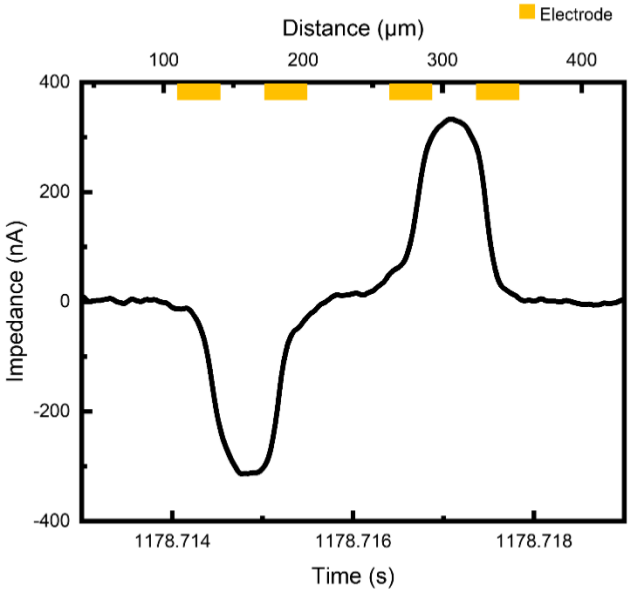

Fig. S4. Demonstration of real-time impedance signals of a droplet, when passing the electrical screening region in the microfluidic channel of the SELECTS. The replacement of low conductive droplet to the original non-conductive oil leading a change of impedance (the peak value indicates the droplet is experiencing the strongest electric field). The rotational symmetric double-peak profile of impedance signals indicates the impedance differentiation between two pairs of electrodes.

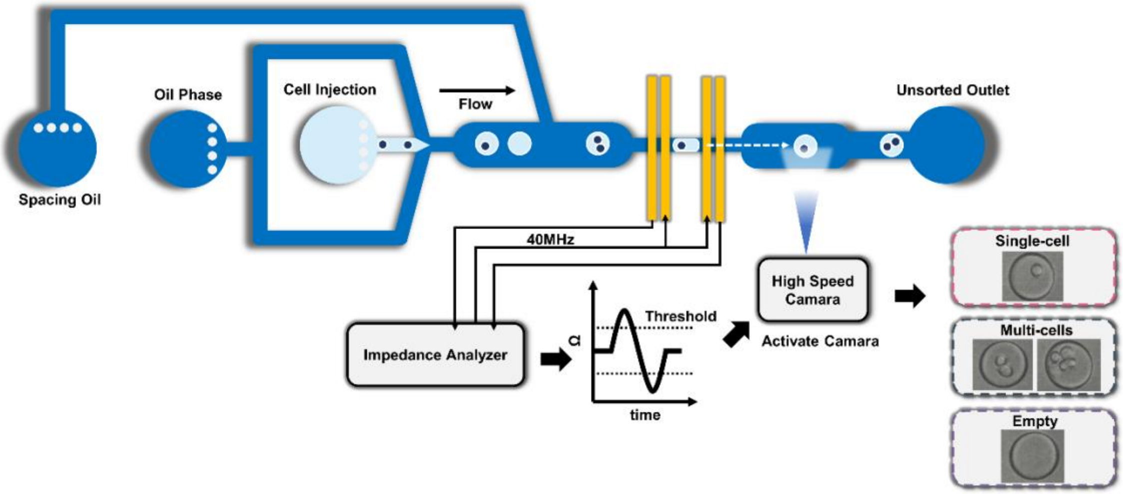

Fig. S5. Schematic of the real-time electrical-activated imaging system that can correspond every impedance signal to respective droplets for validation purpose. When a droplet floats through the impedance screening region, it causes a change of impedance called a droplet event as described in fig. S1. A threshold is properly set to capture all droplet events and triggers the high-speed camara at the frame rate of 1000 fps. The impedance signals are record in real-time and processed in MATLAB to match with the droplet images shotted by the high-speed camara.

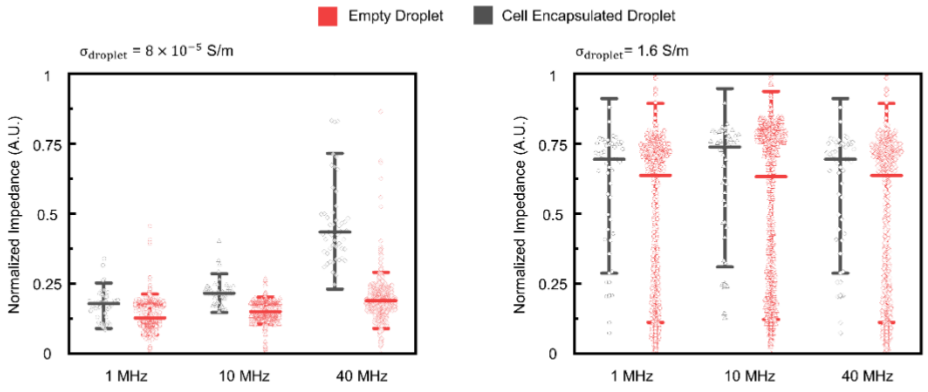

Fig. S6. Experimental results of impedance screening for empty and cell-encapsulated droplets in low and high conductivity medium. For the low conductive medium in droplets (left), increasing probe frequency from 1 MHz to 40 MHz enhances the discrimination between empty (red) and cell-encapsulated (black) droplets. In the contrary, it is unable to classify empty and cell-encapsulated droplets in the high conductive medium (right). Data are presented as mean ± s.d.

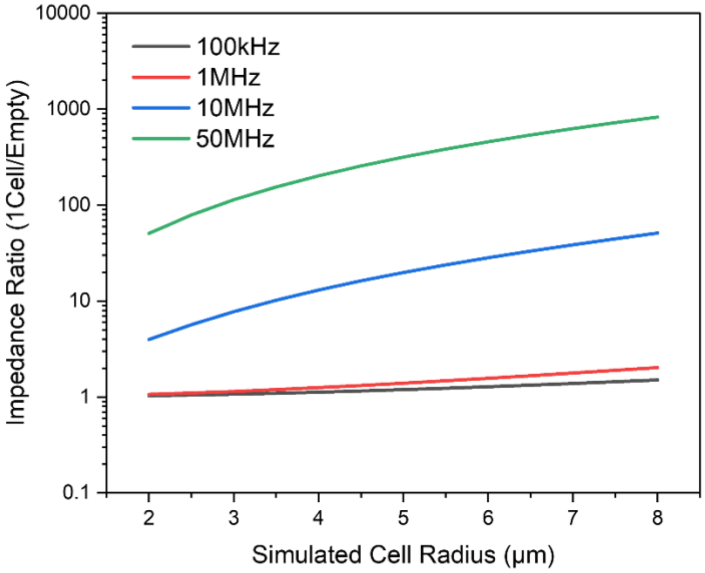

Fig. S7. Simulated impedance ratio by varying cell radius of the cells encapsulated in droplets. The impedance ratio is defined as the fraction of the impedance of 1-cell in droplets over of empty droplets. The impedance ratio increases when increasing the cell radius from 100 kHz to 50 MHz. However, with the probe frequency that is higher than 10 MHz, the impedance ratio is sufficiently significant (> 10) regardless of cell radius.

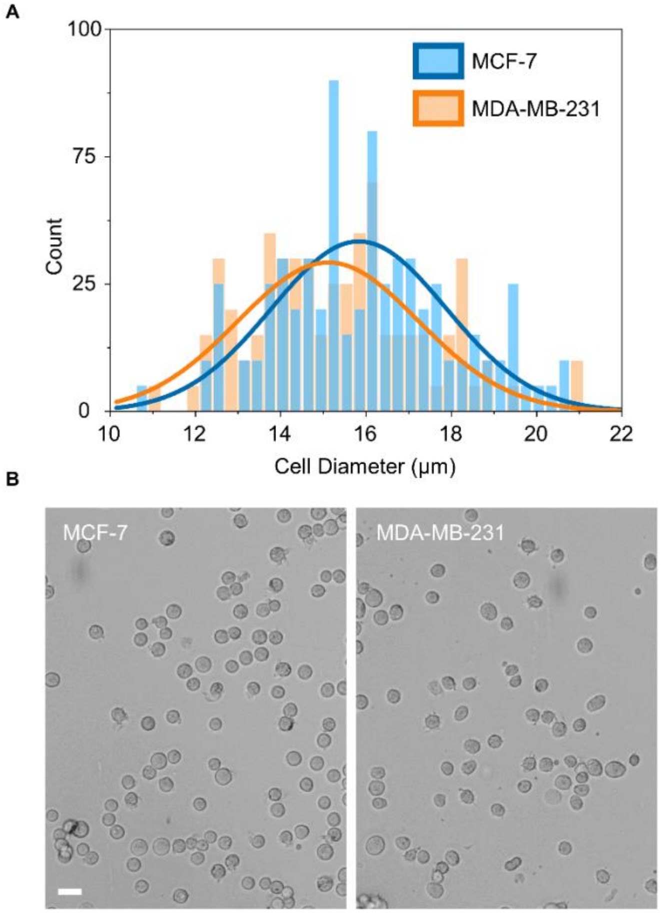

Fig. S8. Cell size measurement of different cell lines: MCF-7 and MDA-MB-231. (A) The histogram indicates cell diameter distribution of MCF-7 (blue) and MDA-MB-231 (orange), and shows that both cell lines have similar cell diameter distribution. (B) Microscopic images for cell diameter measurement. The scale bar is 20 μm.

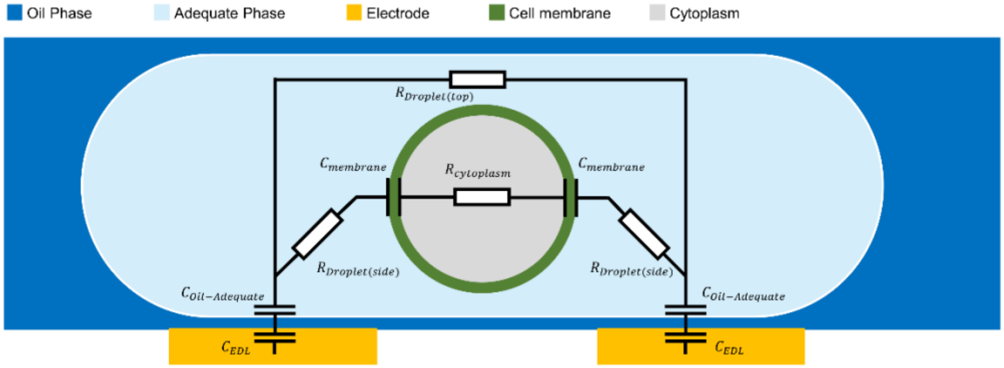

Fig. S9. Schematic of an equivalent circuit model for single-cell encapsulation in droplets on a pair of electrodes of the electrical-based droplet screening region in SELECTS. Since the electric conductivity of adequate medium in droplets is low (8e^{-5} S/m), varying the length of equivalent resistance ($R_{droplet(side)}$) can be induced a significant impedance change. This is proofed in experiment results for differentiating single-cell and multi-cells droplets.

Table S1. Simulation parameters.

Parameter	Value
$h_{Channel}$	20 µm
$w_{Channel}$	20 µm
$w_{electrode}$	20 µm
d_{cell}	10 µm
ε_{medium}	80
σ_{medium}	1.6 / 8e^{-5} S/m
$d_{membrane}$	7 nm
$\varepsilon_{membrane}$	5
$\sigma_{membrane}$	1e^{-7} S/m
$\varepsilon_{cytoplasm}$	60
$\sigma_{cytoplasm}$	0.5 S/m
ε_{oil}	5.8
σ_{oil}	5e^{-9} S/m

Supplementary movies

Movie S1. Demonstration of the SELECTS system. Note that the flow rate of droplet has been reduced for screen-recording.

Movie S2. Sorting all droplets to the target outlet. The video is to demonstrate the acoustic radiation force acting on droplets is identical regardless of their contents.

Movie S3. Sorting single-cell encapsulated droplets to the target outlet. The video is to demonstrate sorting single-cell droplets from empty droplets as conventional active sorting techniques.

Movie S4. Demonstration of multi-cells encapsulated droplets rejection. The video is to demonstrate a novel selection of single-cell droplets from empty and multi-cells droplets which has yet been reported in conventional active sorting techniques.
